# Unveiling altered CD8 T-cell metabolism and homeostatic proliferation behind a low CD4/CD8 ratio in ART-suppressed HIV individuals with normal CD4 recovery

**DOI:** 10.3389/fimmu.2025.1617674

**Published:** 2025-09-08

**Authors:** Vanesa Garrido-Rodríguez, Ángel Bulnes-Ramos, Israel Olivas-Martínez, María del Mar Pozo-Balado, Francisco Tejerina-Picado, Félix Gutiérrez, Cristina Marco-Sánchez, Juan Manuel Tiraboschi, Antonia Castillo-Navarro, Enrique Bernal, Maria C. Garcia-Guerrero, Maria C. Puertas, Joaquim Peraire, Anna Rull, Javier Martinez-Picado, Yolanda María Pacheco

**Affiliations:** ^1^ Servicio de Inmunología, Instituto de Biomedicina de Sevilla, Instituto de Biomedicine de Sevilla (IBiS)/Hospital Universitario Virgen del Rocío/ Consejo Superior de Investigaciones Científicas CSIC/Universidad de Sevilla, Sevilla, Spain; ^2^ Servicio de Microbiología Clínica y Enfermedades Infecciosas, Hospital General Universitario Gregorio Marañon, Madrid, Spain; ^3^ Centro de Investigación Biomédica en Red de Enfermedades Infecciosas (CIBERINFEC), Instituto de Salud Carlos III, Madrid, Spain; ^4^ Hospital General de Elche & Universidad Miguel Hernández, Alicante, Spain; ^5^ Centro Nacional de Epidemiología, Instituto de Salud Carlos III, Madrid, Spain; ^6^ Infectious Diseases Department, Hospital Universitari de Bellvitge, Hospitalet de Llobregat, Barcelona, Spain; ^7^ Hospital Universitario Virgen de la Arrixaca, Instituto Murciano de Investigación Biosanitaria (IMIB), Murcia, Spain; ^8^ Hospital General Universitario Reina Sofía, Universidad Miguel Hernández de Elche, Universidad de Murcia, Murcia, Spain; ^9^ IrsiCaixa AIDS Research Institute, Hospital Universitari Germans Trias i Pujol, Barcelona, Spain; ^10^ Institut Investigació Sanitària Pere Virgili (IISPV), Tarragona, Spain; ^11^ Hospital Universitari de Tarragona Joan XXIII (HJ23), Tarragona, Spain; ^12^ Universitat Rovira i Virgili (URV), Tarragona, Spain; ^13^ University of Vic-Central University of Catalonia (UVic-UCC), Vic, Spain; ^14^ Catalan Institution for Research and Advanced Studies (ICREA), Barcelona, Spain; ^15^ Universidad Loyola Andalucía, Facultad de Ciencias de la Salud, Sevilla, Spain

**Keywords:** HIV-infection, CD4/CD8 ratio, nadir-CD4 T cell, immunometabolism, viral reservoir, thymic output

## Abstract

**Background:**

People living with chronic HIV (PLWH) show immune dysfunction, despite viral suppression and normal CD4 recovery, particularly those with low CD4/CD8 ratios. Subjacent cellular alterations of such a reliable marker of clinical progression remain elusive.

**Methods:**

Categorization by CD4/CD8 ratio after three year of therapy (R < 0.8/R > 1.2, *n* = 28/*n* = 24) and *post-hoc* reclassification by nadir-CD4 (*N* ≤ 350/*N* > 350) were performed in PLWH achieving viral suppression and CD4 ≥ 500. CD4 T cell–associated viral reservoir, as well as metabolism-related gene expression, glucose uptake ability, relative telomere length (RTL), and thymic output for CD4 and CD8 T cells, were determined.

**Results:**

Patients with a CD4/CD8 ratio < 0.8 exhibited reduced CD8 T-cell glucose uptake ability after stimulation (*p* = 0.007) and trends to shorter RTL (*p* = 0.093) and to larger CD4-associated viral reservoir (*p* = 0.068) than *R* > 1.2. Differently, patients with nadir ≤350 exhibited altered CD4 and CD8 T-cell expression of metabolism-related genes, although no differences in glucose uptake ability, and shorter RTL in both cell subsets, but similar viral reservoir to patients with nadir >350. Remarkably, viral reservoir and both CD4 and CD8 thymic output showed inverse associations (*r* = −0.623, *p* = 0.01 and *r* = −0.661, *p* = 0.038, respectively).

**Conclusion:**

A low CD4/CD8 ratio in chronic PLWH stands on a larger viral reservoir in CD4 T cells and metabolic alterations in CD8 T cells, probably related to its exhaustion and compromised effector functionality, and thymic output could contribute to such alterations. Patients with lower nadir-CD4 showed a resting-like CD4 phenotype and a metabolically active CD8 subset, without further viral reservoir extension. Persistence of low CD4/CD8 ratio and low nadir-CD4 counts seems to rely on different immune damage.

## Introduction

People living with HIV (PLWH) exhibit immune dysfunction even under suppressive antiretroviral therapy (ART) (1). Furthermore, despite achieving normal CD4 T-cell counts, immune damage persists, as reflected by increased comorbidities and risk of death observed in this population (2). The CD4/CD8 T-cell ratio has emerged as a more accurate prognostic marker than CD4 counts alone. We recently showed that PLWH on successful ART with normal CD4 recovery but keeping a low CD4/CD8 ratio specifically involves residual inflammation before ART and persistent CD8 T-cell senescence after ART (3). However, the cellular mechanisms driving such immune dysfunction remain poorly understood (4). As for the nadir CD4 count, another well-documented prognostic marker, the mechanisms underlying its clinical relevance are also unknown (5).

Cellular metabolism significantly affects T-cell function and immunity (6). Naïve T cells rely on oxidative phosphorylation (OXPHOS), but upon activation, shift to glycolysis and increase OXPHOS to promote rapid proliferation and to meet energy demands (6). Moreover, HIV preferentially targets metabolically active CD4 T cells (7). In fact, metabolite carriers, such as GLUT1, favor HIV-1 replication within these cells (8). Glycolysis inhibition reduces HIV-1 production, while mTOR, a glycolysis mediator, contributes to HIV latency in CD4 T cells (9). Additionally, the size of the HIV reservoir correlates with disease progression in treatment discontinuation studies and has been proven as a predictor of the immunovirological response to early ART as well as of the magnitude of the viral rebound (10).

Homeostatic proliferation, driven by thymic naïve T-cell output, influences the viral reservoir size (11, 12). Thymic function also impacts immune recovery during ART (13), with previous evidence linking thymic failure to disease progression during HIV infection (14). Indeed, we previously demonstrated a key role of the pre-therapy thymic function in preserving the peripheral CD4/CD8 ratio in this context (3, 15).

Here, we aimed to study whether T-cell metabolic activity, their peripheral proliferation, and the extension of the T cell–associated HIV viral reservoir could be related to the persistence of low CD4/CD8 ratios in PLWH achieving ART suppression and normal CD4 recovery. We also explored whether these factors could be similarly shaped by having initiated ART with a low nadir CD4 count.

## Methods

### Subjects and study design

Post-ART peripheral blood mononuclear cells (PBMCs) and pre-therapy plasma samples were obtained from the Spanish CoRIS Cohort (16) and kindly provided by its HIV BioBank. Samples were processed under standardized procedures and frozen upon reception: PBMCs in liquid nitrogen and plasma at −80°C. Exclusion criteria were (a) no ART or ART initiation before 2010, (b) detectable viral load after the first 6 months of ART, (c) less than 3 years under ART, (d) detectable viral load during the first 3 years of ART (excluding the initial 6 months), (e) no baseline sample, (f) no follow-up sample (3–3.5 years post-ART), and (g) CD4 T-cell counts below 500 cells/mm^3^. From 15,509 CoRIS participants, 123 met the eligible criteria. We analyzed individuals with a CD4/CD8 T-cell ratio < 0.8 (*n* = 24; *R* < 0.8) or > 1.2 (*n* = 28; *R* > 1.2), corresponding to CD4/CD8 ratio values of 1 ± 0.2. Included patients were further classified by nadir-CD4 counts into ≤350 cells/mm^3^ (*n* = 24; *N* ≤ 350) or >350 cells/mm^3^ (*n* = 28; *N* > 350), as the critical threshold for AIDS diagnosis, monitoring treatment interruptions, and identification of late presentation. The nadir CD4 count was defined as the lowest CD4 T-cell value registered before ART initiation and was obtained from the clinical database of the CoRIS cohort. The flowchart is detailed in **Supplementary Figure S1**. Written informed consent was obtained from all participants prior to enrolling in the CoRIS cohort, and the project was approved by the Research Ethics Committee of University Hospitals Virgen Macarena-Virgen del Rocío. Clinical and demographic data were retrieved from CoRIS. All determinations were performed at follow-up, except for soluble thymosin α1 levels, measured at baseline.

### CD4 and CD8 T-cell isolations

Magnetic T-cell isolation was performed from PBMCs following the manufacturer’s instructions (StemCell). First, CD4+ T cells were positively enriched, while CD8+ T cells were isolated by negative selection from the CD4-depleted fraction. Purity was verified in every sample by flow cytometry, and only samples with >95% purity were used.

### Metabolism-related gene expression quantification

Total RNA was purified from isolated CD4 or CD8 T cells using the RNeasy Mini Kit (Qiagen, Hilden, Germany). A minimum of 500,000 cells was preserved in TriPure isolation reagent (Sigma-Aldrich, St. Louis, USA) and treated with DNase. 50 ng of RNA was retrotranscribed into cDNA using the iScript cDNA Synthesis Kit (Bio-Rad). Gene expression was quantified by RT-PCR with TB Green Premix Ex Taq (Takara, Kusatsu, Japan). Specific primers were designed using Primer3 Input 4.1.0, and their sequences are detailed in **Supplementary Table S1**. cDNA was amplified under the following conditions: 50°C for 2 min and 95°C for 2 min, followed by 40 cycles of 95°C for 15 s and 56°C–60°C (2.2°C/s with a delay of 8 cycles) for 1 min and, finally, a melting curve of 95°C for 1 min, followed by 40°C for 1 min, then 70°C for 1 s and 95°C (continuous). qPCR was performed in a LightCycler 480 System (Roche, Copenhagen, Denmark). Gene expression was normalized by the expression of the single-copy gene β-globin. Relative quantification was determined using the 2^-ΔΔCt^ method.

### *In-vitro* glucose uptake assay

Glucose uptake was measured in 20,000 isolated CD4 and CD8 T cells seeded for 1h in RPMI containing 10% heat-inactivated fetal bovine serum (FBS), 100 µg/ml penicillin/streptomycin, and 2 mM glutamine. Cells were stimulated for 1h with 50 ng/ml PMA and 1 µg/ml ionomycin. For glucose uptake determination, cells were incubated in DPBS without glucose and FBS. After three washes with DPBS, cells were collected by centrifugation, and glucose uptake was determined in the cell lysate using the Glucose Uptake-Glo™ Assay Kit (Promega). This luminescence-based assay quantifies the intracellular accumulation of 2-deoxyglucose-6-phosphate (2DG6P), and the resulting signal is directly proportional to the glucose uptake capacity of the cells. Absolute glucose uptake concentrations (in nM) were calculated using a standard curve generated from known concentrations of the glucose analog provided in the kit.

### Thymic function determination

The sj/β TRECs ratio was quantified using droplet digital PCR (ddPCR) on genomic DNA extracted from isolated CD4 and CD8 T cells. Signal-joint (sj) and DβJβ T-cell receptor excision circles (β-TRECs) were amplified in parallel using specific primers and probes detailed elsewhere (17), and the ratio between sj and β-TRECs was calculated to estimate thymic output, as this reflects the extent of intrathymic proliferation. All reactions were performed using a QX200 ddPCR system (Bio-Rad) following protocols previously described elsewhere (17).

Thymosin α1 (TMα1) was quantified in pre-therapy plasma samples using the Human Thymosin Alpha 1 Elisa kit (MyBiosurce^®^), following the manufacturer’s instructions.

### Relative telomere length determination

DNA from stored CD4 and CD8 T cells was extracted using the Omega BIO-TEK, E.Z.N.A blood DNA Mini Kit. For each reaction, 60 ng of DNA were used to amplify telomeres and the single-copy gene β-globin (see primer sequences in **Supplementary Table S1**) by qPCR (17) using a Light Cycler 480 (Roche, Copenhagen, Denmark). Relative telomere length (RTL) was calculated as the telomere copies/single-copy gene ratio.

### Viral reservoir quantification

CD4+ T cells were lysed overnight at 55°C in a buffer containing 0.1% Triton-X100 and 400 µg/ml Proteinase K in 10 mM Tris-HCl, followed by 5 min inactivation of the proteinase at 95°C and further centrifugation at 10,000*g* for 1 min to clarify the sample from magnetic beads. Total HIV DNA was measured in cell lysates by quantification of GAG or 1LTR HIV target genes by ddPCR as described (18), and results were normalized based on parallel determination of the cell gene RPP30.

### Data analysis

Quantitative variables are expressed as median and interquartile range [IQR]. Cross-sectional comparisons between groups were performed using the Mann–Whitney *U* test, while longitudinal comparisons were performed using the Wilcoxon signed-rank test. Categorical variables were recorded as the number of cases and percentages, with comparisons among groups using the χ^2^ or Fischer’s exact test. Associations between variables were explored by Spearman’s rho correlation coefficient, excepting collinearity analysis that was assessed by Pearson’s correlation and considered significant when *p* < 0.05. Analyses were performed using Statistical Package for the Social Sciences software (SPSS 25; IBM SPSS), and atypical values were detected by Dixon’s Q test using the *Outliers* package in R. Dot plots and heat maps were created with the *ggplot2* package in R.

## Results

### Characteristics of the study cohort

Patients with CD4/CD8 ratios of *R* < 0.8 and *R* > 1.2 presented similar demographic and clinical characteristics, including age, sex, HCV/HBV coinfections, time from diagnosis to ART initiation, ART duration/composition, baseline viral load, and time to undetectable viral load (**Table 1**). However, patients with *R* < 0.8 exhibited lower nadir-CD4 (296 [250–352] vs. 447 [356–607], *p* < 0.0001), baseline CD4 counts (317 [261–377] vs. 494 [399–632], *p* < 0.0001) and follow-up CD4 counts (663 [560–749] vs. 904 [753–1107], *p* < 0.0001) compared to *R* > 1.2. The CD4/CD8 ratio was lower in *R* < 0.8 patients at baseline (0.38 [0.24–0.48] vs. 0.78 [0.51–1.01], *p* = 0.001), and follow-up (0.63 [0.58–0.73] vs. 1.53 [1.33–1.78], *p* < 0.0001). CD8 T-cell counts were higher in R < 0.8 patients at follow-up (985 [886–1346] vs. 607 [525–695], *p* < 0.0001) and showed a trend at baseline (949 [756–1463] vs. 738 [593–808], *p* = 0.066). See **Supplementary Figure S1** and **Table 1** for details.

**Table 1:** Characteristics of the study subjects classified according to CD4/CD8 ratio at follow-up or their nadir-CD4 values.

Parameter	Entire cohort *n* = 52	CD4/CD8 ratio			Nadir-CD4		
		*R* < 0.8 *n* = 24	*R* > 1.2 *n* = 28	*p*-value	*N* ≤ 350 *n* = 24	*N* > 350 *n* = 28	*p*-value
**Age (years)**	39[33–46]	39 [33–50]	39 [31–44]	0.335	37 [29–44]	42 [35–47]	0.078
**Males, *n* (%)**	52(100)	24 (100)	28 (100)	1	24 (100)	28 (100)	1
**Diagnosis to treatment time (months)**	4[2–18]	5.5 [1.3–15.5]	4 [2–21]	0.876	3.5 [0.3–12.8]	5.0 [2.0–21.0]	0.070
**HCV antibodies, *n* (%)**	3 (5.8)	3 (12,5)	0	0.128	2 (8.3)	1 (3.6)	0.244
**ART duration (months)**	39[37–40]	40 [37–41]	39 [37–39]	0.095	39 [37–40]	39 [38–40]	0.576
**ART composition, *n* (%)**				0.231			0.799
2NRTI + 1NNRTI	36 (69.2)	14 (58.3)	22 (78.6)		17 (70.8)	19 (67.9)	
2NRTI + 1PI	1 (1.9)	1 (4.2)	0		0	1 (3.6)	
2NRTI + 1INSTI	10 (19.2)	7 (29.2)	3 (10.7)		5 (20.8)	5 (17.9)	
Other*	5 (9.6)	2 (8.3)	3 (10.7)		2 (8.3)	3 (10.7)	
**Log viral load at baseline (copies/ml)**	4.69[4.27–5.07]	4.50 [4.15–5.02]	4.68 [3.79–5.20]	0.664	4.69 [4.14–5.17]	4.71 [4.29–4.99]	0.710
**Time to reach viral load undetectability (months)**	3[2–5]	3.5 [2–5]	3 [2–5]	0.896	3 [2–5]	3 [2–5]	0.730
CD4 (cell/mm^3^)							
Nadir	362[285–475]	296 [250–352]	447 [356–607]	**< 0.0001**	275 [224–304]	466 [397–612]	**< 0.0001**
Baseline	388[303–574]	317 [261–377]	494 [399–632]	**< 0.0001**	303 [230–361]	479 [391–623]	**< 0.0001**
Follow-up	779[610–1015]	663[560–749]	904 [753–1107]	**< 0.0001**	643 [553–855]	893 [738–1092]	**< 0.0001**
ΔCD4**	347[232–497]	306[230–485]**^#^**	402[256–498]**^#^**	0.481	331[233–434]**^#^**	402[215–507]**^#^**	0.950
CD8 (cell/mm^3^)							
Baseline	808[602–1205]	949 [756–1463]	738 [593–808]	0.066	903 [628 –1463]	794 [593–1025]	0.578
Follow-up	741[606–1077]	985 [886–1346]	607 [525–695]	**< 0.0001**	918 [696–1234]	677 [578–865]	**0.018**
ΔCD8**	−86[−371 ± 137]	−1[−354 ± 320]**^#^**	−119[−383 ± 54]**^#^**	0.126	−52[−372 ± 258]	−119[383 ± 87]	0.538
CD4/CD8 ratio							
Baseline	0.50[0.29–0.80]	0.38 [0.24–0.48]	0.78 [0.51–1.01]	**0.001**	0.36 [0.24–0.58]	0.68 [0.45–0.82]	**0.031**
Follow-up	1.23[0.65–1.58]	0.63 [0.58–0.73]	1.53 [1.33–1.78]	**< 0.0001**	0.67 [0.58–1.18]	1.36 [1.21–1.68]	**< 0.0001**
ΔCD4/CD8**	0.44[0.25–0.83]	0.25[0.21–0.34]**^#^**	0.82[0.69–1.1]**^#^**	**< 0.0001**	0.31[0.23–0.48]**^#^**	0.82[0.29–1.13]**^#^**	**0.013**

Quantitative variables expressed as median [IQR]; statistical analyses between groups were performed using a non-parametric Mann–Whitney test. Qualitative variables expressed as number of cases (%); statistical analyses were performed using χ2 or Fischer’s exact tests. No differences between groups were found in educational level. Statistical significance is highlighted in bold.

HCV, Hepatitis C virus; ART, antiretroviral treatment; NRTI, nucleoside-analog reverse transcriptase inhibitors; NNRTI, non-nucleoside reverse transcriptase inhibitors; PI, protease inhibitors; INSTI, integrase strand transfer inhibitors.

*Other ART composition includes ATV+MCV+RTV, FTC+TAF, DTG+RPV, ATV+TRV+3TC, DRV+RTV

**ΔCD4, ΔCD8 and ΔCD4/CD8 ratio represents the difference between baseline and follow-up values CD4, CD8 and CD4/CD8 ratio values, respectively.

# indicates *p* < 0.01 when baseline versus postART values are compared, applying a Wilcoxon’s signed-rank test.

When reclassified by nadir-CD4, patients with *N* ≤ 350 tended to be younger that those with *N* > 350 (37 [29–44] vs. 42 [35–47] years, *p* = 0.078) and exhibited a trend towards shorter diagnosis-to-treatment time (3.5 [0.3–12.8] vs. 5.0 [2.0–21.0] months, *p* = 0.070). Most *R* < 0.8 patients belonged to the group of *N* ≤ 350 when reclassified (18/24, 75%). Nevertheless, the CD4/CD8 ratio and nadir-CD4 showed a non-collinear association between them (Pearson’s coefficient, *r* = 0.328). Similarly to CD4/CD8 ratio comparisons, patients with nadir *N* ≤ 350 had lower baseline (303 [230–361] vs. 479 [391–623], *p* < 0.0001) and follow-up (643 [553–855] vs. 893 [738–1092, *p* < 0.0001) CD4 counts. The CD4/CD8 ratio was lower in *N* ≤ 350 at baseline (0.36 [0.24–0.58] vs. 0.68 [0.45–0.82], *p* = 0.031) and follow-up (0.67 [0.58–1.18] vs. 1.36 [1.21–1.68], *p* < 0.0001). However, only follow-up CD8 counts, not baseline, differed (918 [696–1234] vs. 677 [578–865], *p* = 0.018).

Of note, in both cohorts, the CD4 T-cell counts exhibit similar near doubling post-follow-up compared to the baseline, whereas the longitudinal increases in the CD4/CD8 ratios were statistically different between cohorts, as expected because of the classification criteria. However, these patterns of reconstitution appear to be consistent irrespective of their classification based on CD4/CD8 ratios or nadir-CD4.

### Altered CD8 T-cell glucose metabolism associated with low CD4/CD8 ratio persistence

**Figure 1:**
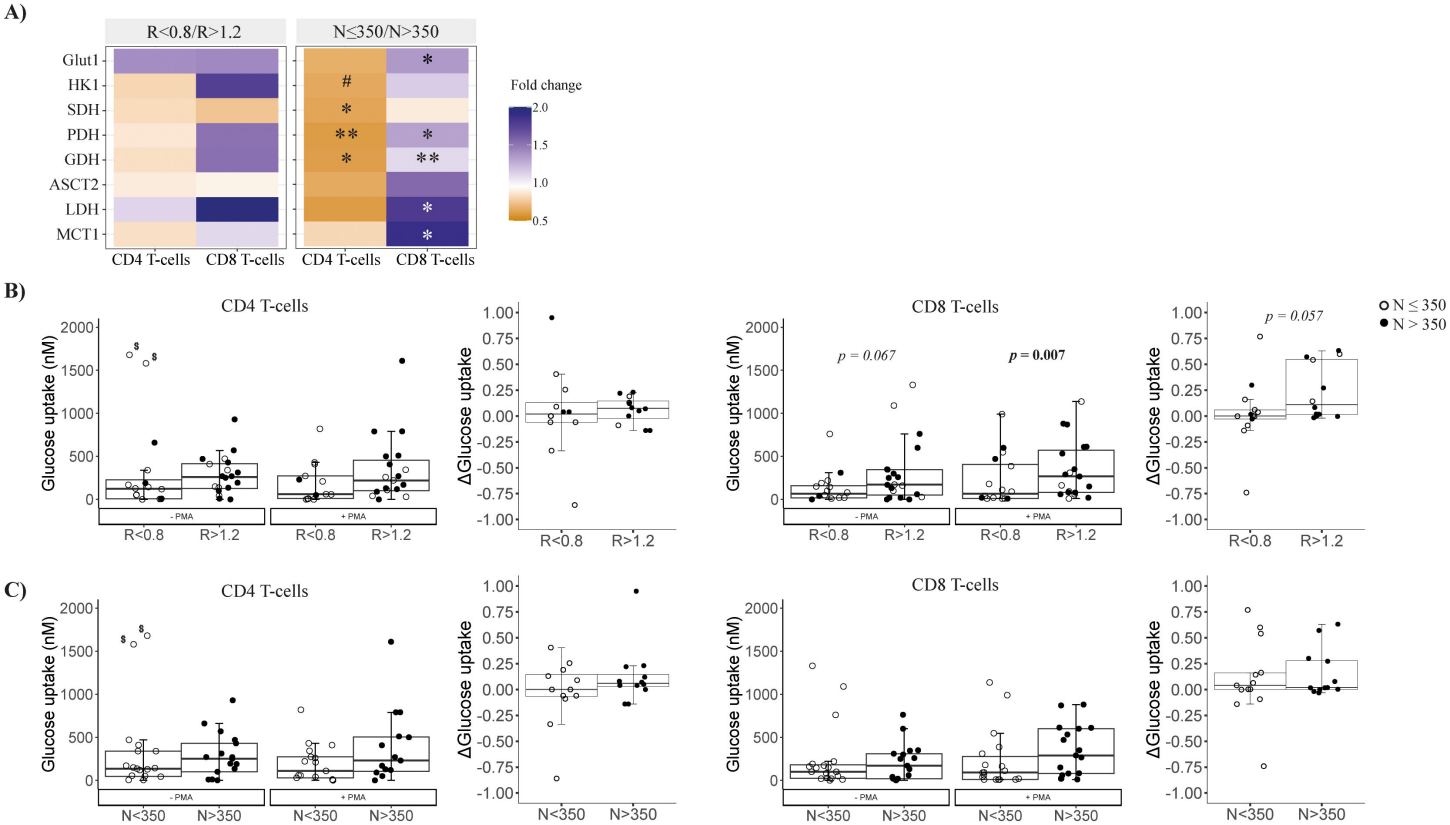
Metabolic characterization of isolated CD4 and CD8 T cells at follow-up. **(A)** Metabolism-related gene expression by CD4 and CD8 T cells at follow-up according to CD4/CD8 ratio (left) or nadir-CD4 (right); differences between groups are represented by the color legend as a fold change (*R* < 0.8/*R* > 1.2 or *N* ≤ 350/*N* > 350). Mann–Whitney tests were performed to compare the distribution of the variables between groups *R* < 0.8 versus *R* > 1.2 or *N* ≤ 350 versus *N* > 350; when statistically significant, *p*-values are indicated as ∗ (*p* < 0.05) or ∗∗ (*p* < 0.01). *P*-values between 0.1 and 0.05 are indicated as #. **(B, C)** Functional characterization of the glucose uptake ability of isolated CD4 and CD8 T cells at the steady-state and after PMA stimulation (left plots of each panel) and Δ glucose uptake (difference between estimulated and steady state glucose uptake; right plots of each panel) comparing groups according to CD4/CD8 or nadir-CD4, respectively. ^$^These values were detected as outliers by Dixon’s Q test; excluding them, *R* < 0.8 versus *R* > 1.2, *p* = 0.043, while *N* ≤ 350 versus *N* > 350, *p* = 0.140. Comparisons were performed between groups *R* < 0.8 versus *R* > 1.2 in each condition (Mann–Whitney *U* test) and longitudinally –PMA versus +PMA (Wilcoxon signed-rank test). Sample size: *R* < 0.8, *n* = 18 and *R* > 1.2, *n* = 19. MCT1, Monocarboxylate transporter; HK1, Hexokinase 1; SDH, Succinate dehydrogenase; GDH, Glutamate dehydrogenase; Glut1, Glucose transporter 1; PDH, Pyruvate, dehydrogenase; ASCT2, Alanine Serine Cysteine transporter 2; LDH, Lactate dehydrogenase.

We analyzed genes related to energy metabolism in isolated CD4 and CD8 T cells: glycolysis (Glut1 and HK1), the Krebs cycle/OXPHOS (SDH and PDH), glutaminolysis (GDH and ASCT2), and lactic acid fermentation (LDH and MCT1). Details and statistical comparisons are in **Supplementary Table S2**. Gene expression in CD8 T cells seemed higher in *R* < 0.8 patients compared to *R* > 1.2 (fold change ~2), but differences were not significant (**Figure 1A**). Whereas functional assays showed that CD4 T cells did not differ between groups, neither in the steady state nor after stimulation (**Figure 1B left**), CD8 T cells from *R* < 0.8 patients in the steady state presented a trend to lower glucose uptake, and, after unspecific stimulation, these cells were not able to capture as much glucose from the media as those from *R* > 1.2 patients (*p* = 0.067 and *p* = 0.007, respectively; **Figure 1B right**).

**Figure 2:**
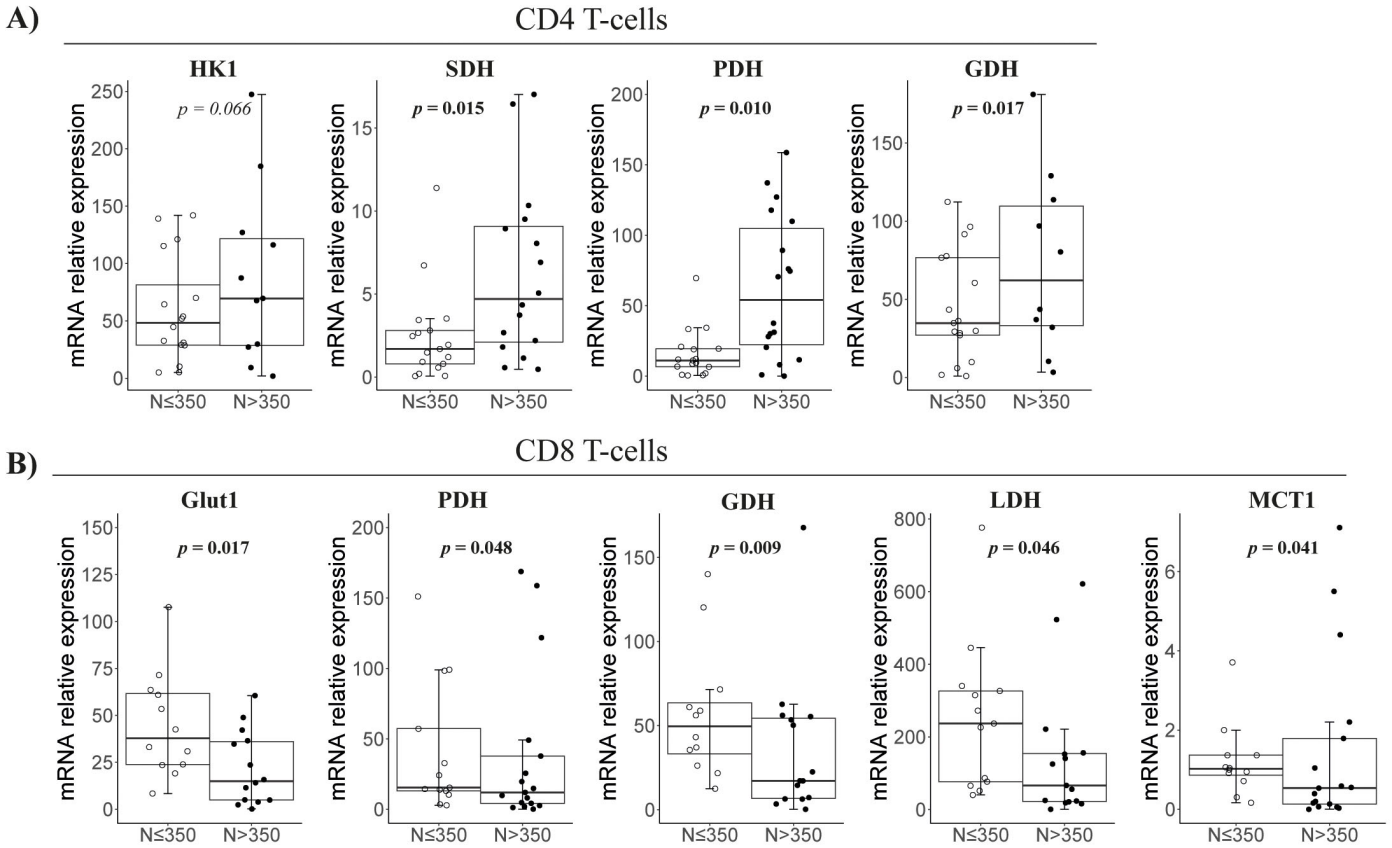
Metabolism-related gene expression in isolated CD4 and CD8 T cells at follow-up according to nadir CD4. Representation of the significant comparisons when comparing nadir-CD4 groups regarding CD4 **(A)** or CD8 T cells **(B)**, respectively. Sample size: *N* ≤ 350, *n* = 18 and *N* > 350, *n* = 19. MCT1, Monocarboxylate transporter; HK1, Hexoquinase 1; SDH, Succinate dehydrogenase; GDH, Glutamate dehydrogenase; Glut1, Glucose transporter 1; PDH, Pyruvate, dehydrogenase; ASCT2, Alanine Serine Cysteine transporter 2; LDH, Lactate dehydrogenase.

By nadir-CD4 classification, CD4 T cells in *N* ≤ 350 patients expressed lower levels of Krebs cycle/OXPHOS-related genes (i.e., SDH, GDH, and PDH, all *p* < 0.02; **Figure 1A**). Conversely, CD8 T cells in *N* ≤ 350 patients exhibited higher expression of genes for the Krebs cycle (GDH and PDH, *p* < 0.05), lactate production (MCT1 and LDH, *p* < 0.05), and glucose uptake (Glut1, *p* = 0.017) (**Figures 2A, B**). However, glucose uptake differences were not significant, neither in CD4 nor in CD8 T cells, and independently of experimental conditions (**Figure 1C**).

### CD4 and CD8 telomere length was only related to nadir, not the CD4/CD8 ratio

**Figure 3:**
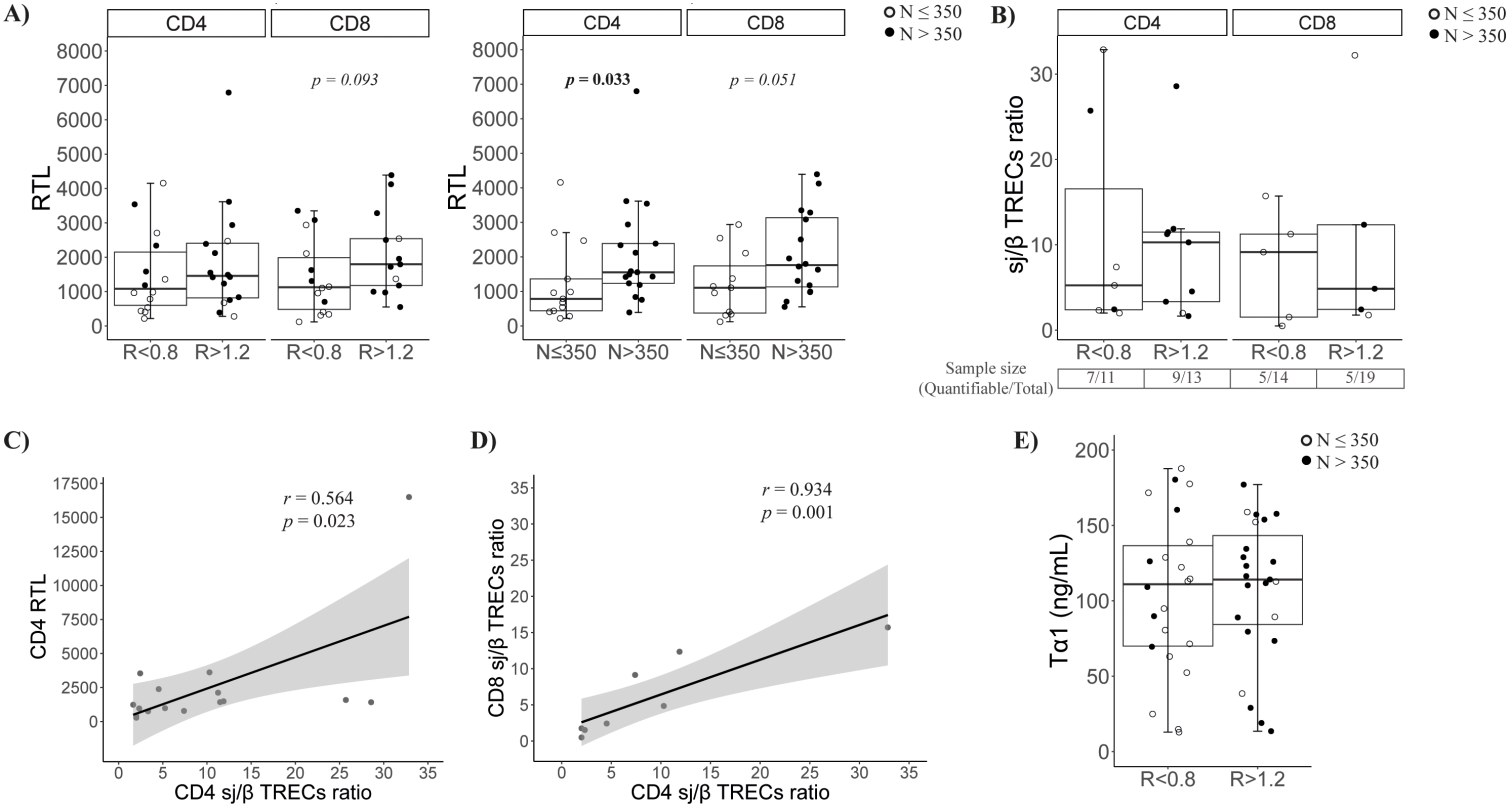
Characterization of the thymic function. **(A)** Relative telomere length (RTL) of CD4 and CD8 T cells at follow-up according to CD4/CD8 ratio (left) or nadir-CD4 (right) classification at follow-up; **(B)** Quantification of sj/β TRECs ratio in isolated CD4 and CD8 T cells according CD4/CD8 ratio (left) or nadir classification (right) at follow-up; **(C)** Association between the sj/β TRECs ratio and the RTL in CD4 T cells; **(D)** Association between CD4 and CD8 sj/β TRECs ratio; **(E)** Soluble thymosin α1 levels at baseline according to CD4/CD8 ratio (left) or nadir-CD4 (right) classification.

We determined RTL in isolated CD4 and CD8 T cells. CD4 T cells showed no differences by CD4/CD8 ratio, but CD8 T cells in *R* < 0.8 tended to have shorter telomeres (*p* = 0.093, **Figure 3A left**). Interestingly, by nadir-CD4 classification, both CD4 and CD8 T cells had shorter telomeres in *N* ≤ 350 (*p* = 0.033 and *p* = 0.051, respectively; **Figure 3A right**).

As a complementary determination of peripheral proliferation, we estimated the thymic output by quantifying the sj/β TRECs ratio. Globally, no differences were observed in CD4/CD8 ratio or nadir-CD4 groups (**Figure 3B**). Nevertheless, the sample size was reduced due to the difficulty of β-TRECs detection (63.6% and 69.2% of CD4 samples; 35.7% and 26.3% of CD8 samples, in *R* < 0.8 and *R* > 1.2, respectively). Remarkably, the CD4 sj/β TRECs ratio directly correlated with their RTL (*r* = 0.564, *p* = 0.023, **Figure 3C**), and the CD4 and CD8 sj/β TRECs ratios were strongly associated (**Figure 3D**). The production of the immunomodulatory hormone thymosin α1 was also quantified in baseline samples, but no significant differences were observed (**Figure 3E**).

### Viral reservoir size was inversely correlated with CD4 and CD8 thymic output

**Figure 4:**
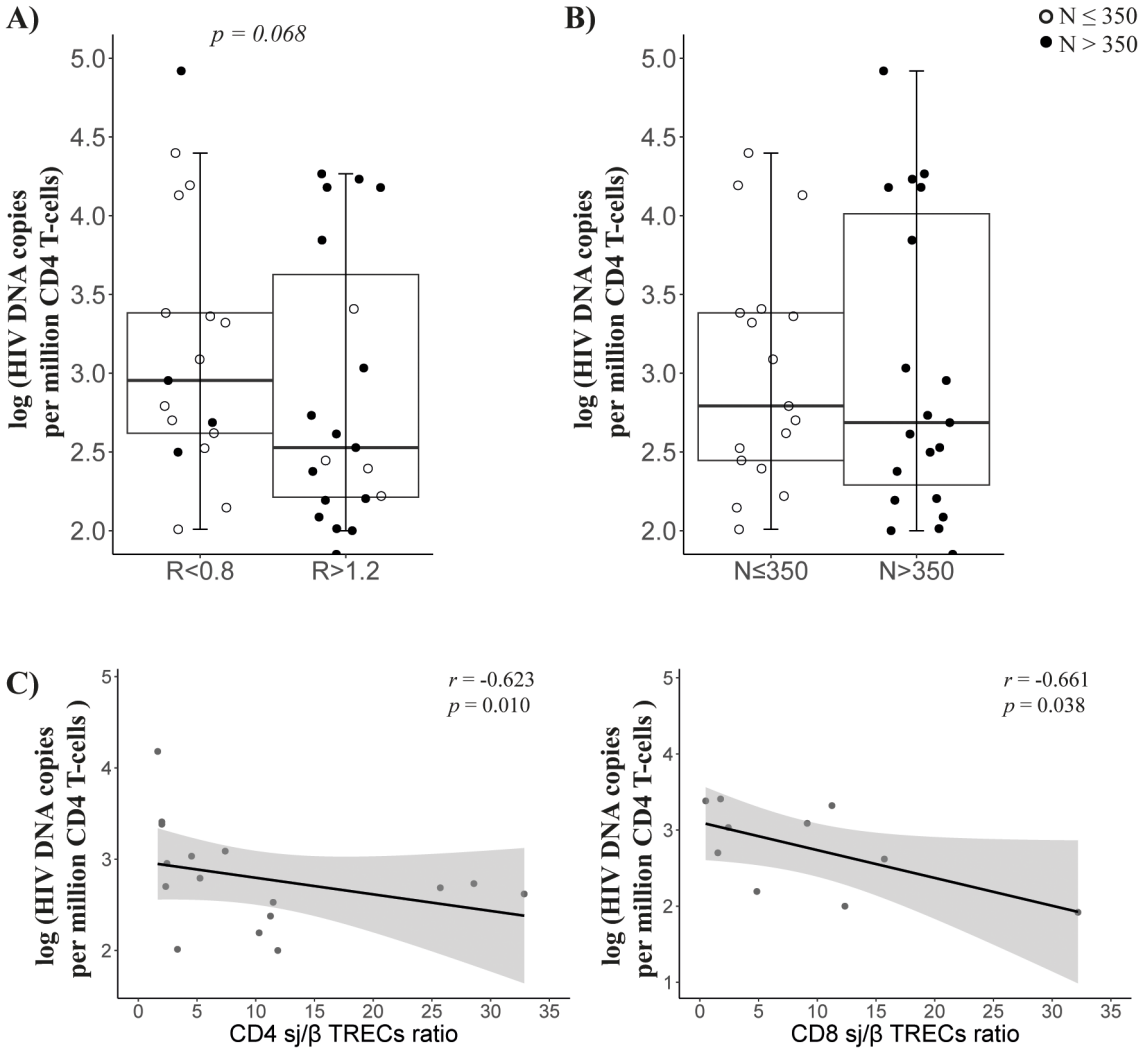
Viral reservoir. Comparison of viral reservoir (HIV DNA copies per million cells) in CD4 T cells between groups according to CD4/CD8 ratio **(A)** or nadir-CD4 **(B)** classification at follow-up; **(C)** Associations between viral reservoir and CD4 (left) or CD8 (right) sj/β TRECs ratio. Sample size: *R* < 0.8, *n* = 17 and *R* > 1.2, *n* = 22; *N* ≤ 350, *n* = 19 and *N* > 350, *n* = 20.

The viral reservoir size, measured as HIV DNA copies per million CD4 T cells, tended to be higher in *R* < 0.8 patients (*p* = 0.068; **Figure 4A**) than in *R* > 1.2 patients, but there was no difference among nadir-CD4 groups (**Figure 4B**). Notably, viral reservoir size inversely correlated with the sj/β TRECs ratio in CD4 and CD8 T cells (*r* = −0.623, *p* = 0.01 and *r* = −0.661, *p* = 0.038, respectively; **Figure 4C**).

## Discussion

Immune dysfunction persists in PLWH with a low CD4/CD8 ratio, despite successful ART and normal CD4 recovery. However, the mechanisms driving ratio inversion remain largely unexplored. Here, we report that PLWH with low CD4/CD8 exhibited reduced glucose uptake ability of CD8 T cells, coupled with trends towards shorter telomere length in this subset and a larger viral reservoir in CD4 T cells. Notably, our findings suggest a potential link between the CD4-associated viral reservoir and thymic output in this context. Additionally, in patients with lower nadir-CD4, we identified a resting-like CD4 phenotype and a metabolically active CD8 subset, without further viral reservoir extension, as differential characteristics.

Regulation of energetic metabolism is crucial for T-cell function and likely impacts the course of HIV infection. Although mitochondrial metabolism also has a crucial role in T-cell homeostasis and function, we did not have access to more advanced approaches such as the Seahorse XF Analyzer, which would have enabled a more comprehensive assessment of mitochondrial respiration and glycolytic flux. However, we included the study of the gene expression of key mitochondrial enzymes, such as succinate dehydrogenase (SDH) and pyruvate dehydrogenase (PDH), involved in the TCA cycle and OXPHOS. These transcriptional markers provided indirect insight into mitochondrial metabolic states, particularly in the nadir-CD4 stratified groups. Here, we found that PLWH with low CD4/CD8 ratios, despite viral suppression and immune recovery, exhibited reduced glucose uptake in CD8 T cells upon activation, despite no changes in the expression of major metabolism-related genes. *Ex-vivo* studies revealed that glycolysis is essential for HIV-specific CD8 cytotoxicity, yet CD8 T cells from chronic PLWH failed to enhance glycolysis and OXPHOS after TCR stimulation, leading to functional exhaustion (19). Indeed, lower CD4/CD8 ratios were also linked to more severe CD8 exhaustion and enrichment in effector populations (20), reflecting reduced glycolysis as a marker of exhaustion.

Notably, although glucose uptake was significantly reduced in CD8 T cells from individuals with a low CD4/CD8 ratio, no parallel decrease in *Glut1* gene expression was detected. This apparent paradox may result from the complex regulation of glucose uptake, which involves not only transcriptional control but also post-translational modifications, transporter trafficking to the membrane, and subset composition. Furthermore, while GLUT1 is the main glucose transporter in activated T cells, other GLUT family members may also contribute, especially in chronically activated or exhausted cells. Alternatively, statistical power could be limited in such a comparison.

In contrast, lower nadir-CD4 patients showed decreased expression of OXPHOS-related genes in CD4 T cells and increased OXPHOS and Glut1 expression in CD8 T cells, despite no changes in glucose uptake. This suggests a senescent CD4 phenotype and an activated CD8 subset. Senescent cells have been shown to develop a senescent-associated secretory phenotype (SASP), triggering mitochondrial dysfunction in the nearby cells, resulting in increased ROS production, DNA damage, and several proinflammatory cytokines (21), potentially driving CD8 activation and systemic inflammation.

Metabolic demands of T cells are highly dependent on their differentiation status. Indeed, the report by Valle-Casuso et al. (7) has demonstrated the distinct metabolic reprogramming across T-cell subsets, highlighting the need for integrated analyses. In a previous study using the same cohort, we identified differences in CD8 T-cell maturation profiles by mass cytometry (3). In contrast, the current study was designed to assess the global metabolic profile of total CD4 and CD8 T cells as an integrated functional readout. While subset-specific analyses could offer deeper mechanistic insight, they were beyond the scope of this work and remain an important direction for future studies.

Finally, our study was not designed to address that specific question; a group of age- and sex-matched HIV-negative controls would have allowed us to assess whether individuals in the *R* > 1.2 or *N* > 350 groups fully normalize metabolic parameters.

Telomere shortening in PLWH has been widely described (22) and is usually attributed to a sustained immune activation and/or immunosenescence (23). Nonetheless, just a few studies have assessed telomere length in isolated CD4 or CD8 T cells. The trend towards reduced telomere length observed in CD8 T cells from low nadir-CD4 patients supports the hypothesis of a greater cellular replicative senescence against the chronic infection in these patients. Moreover, telomere attrition in CD4 T cells from low nadir CD4 patients may stem from compensatory proliferation during ART-induced CD4 replenishment. Litchterfeld et al. demonstrated that HIV-1–specific CD8 T cells from elite controllers exhibited longer telomeres than progressors, and that was associated with higher levels of constitutive telomerase activity (24), linking telomere attrition to immunological recovery and clinical progression (25).

Thymic output is another driving factor of immunosenescence, which plays a key role in CD4 T-cell homeostasis, although its role in the CD8 T-cell homeostasis in the context of HIV infection has been less studied. Remarkably, it has been previously described as having associations with CD4/CD8 ratio (15), as well as with nadir-CD4 (14), in the context of HIV infection. We quantified the sj/β TRECs ratio as the gold standard for the measure of thymic output; however, it presented technical limitations, mainly regarding the detection of β-TRECs, preventing us from observing the expected differences between study groups. Nevertheless, our data show a coherent association between the RTL in CD4 T cells and their content in sj/β TRECs ratio, suggesting that a lower thymic output would consequently involve higher peripheral proliferation of these cells that would affect CD4 or CD8 T cells differentially.

Interestingly, only patients with lower CD4/CD8 ratios exhibited trends toward a larger viral reservoir. However, we cannot completely rule out the possibility of a similar trend in the nadir-based comparison with a larger sample size. In this sense, Ismail et al. recently supported an inverse correlation between reservoir size and both CD4/CD8 ratio and nadir-CD4 before initiating ART (26). Furthermore, several studies have demonstrated a higher CD4/CD8 ratio after a suppressive ART to be predictive of a lower viral reservoir size (27). Interestingly, we observed negative associations between the size of the viral reservoir and the sj/β TRECs ratio in both CD4 and CD8 T cells, suggesting a potential relationship between the viral reservoir and the thymic output that could be involving the peripheral compensatory proliferation (11), not only for CD4 but also for CD8 T cells. Consistently, in a previous report, naïve CD4 T cells showed higher content of sj-TRECs compared with central memory and effector CD4 T cells, but lower content of HIV proviral DNA (28). However, this phenomenon may also be caused by the reduced expression of CCR5 in naïve T cells (29), leading to a reduced susceptibility to infection in these cells.

A functionally compromised CD8 phenotype may contribute to larger viral reservoirs, which eventually determines the magnitude of exposure to HIV antigens. In fact, the presence of CD8 T cells plays a major role in CD4 functionality and viral reservoir reactivation (30). Using a model of non-human primate HIV infection, Cartwright et al. reported that *in-vivo* CD8 T-cell depletion in SIV-infected Rhesus macaques under ART caused the reactivation of SIV replication and production (30). Recently, it has also been demonstrated that CD8 T cells induce metabolic changes in CD4 T cells, inhibiting glycolysis and OXPHOS metabolic pathways, promoting cell survival, and inducing a quiescent state, which avoids HIV replication and promotes viral latency (31). Reduced thymic output may impair CD8 function, exacerbating CD8 exhaustion and hindering reservoir control. Coherently, we also found an exhausted CD8 T-cell phenotype, along with enhanced intestinal homing imprinting and higher frequencies of antigen-presenting cells associated with the persistence of a low CD4/CD8 ratio (3).

This exploratory, hypothesis-generating study has a limited sample size due to strict selection criteria, which nevertheless allowed for a homogeneous cohort. We cannot exclude the influence of factors such as medication, comorbidities, or viral blips on the results, particularly the metabolism assays and viral reservoir determination. However, clinical parameters related to ART did not differ between groups. Despite the overlap between low CD4/CD8 ratio and low nadir-CD4 patients, we report consistent and differential observations with each classification. Understanding the determinants of these biomarkers is crucial for comprehending their relationship with clinical progression (4). Our data suggest that a persistent low CD4/CD8 ratio and low nadir CD4 would reflect distinct immunological damage that may differentially impact clinical progression and, consequently, deserve specific therapeutic approaches.

While our study analyzed CD4/CD8 ratio and nadir CD4 counts as independent stratification variables, it is plausible that individuals presenting both alterations simultaneously (i.e., *R* < 0.8 and nadir CD4 < 350) may exhibit more pronounced immune-metabolic dysfunction due to a potential additive effect. However, given the limited sample size in our cohort for discordant combinations, such as *R* < 0.8 with *N* > 350 or *R* > 1.2 with *N* < 350 (*n* = 6 in each case in our study cohort), we could not perform these further stratified analyses. Future studies with larger cohorts will be necessary to explore the clinical and biological relevance of combined classifications.

## Data Availability

Data will be made available upon reasonable request to the corresponding author.
