# Supplementary material for: Unveiling altered CD8 T-cell metabolism and homeostatic proliferation behind a low CD4/CD8 ratio in ART-suppressed HIV individuals with normal CD4 recovery
Source: Front Immunol. 2025 Sep 8;16:1617674. doi: 10.3389/fimmu.2025.1617674 (PMC12450686; doi:10.3389/fimmu.2025.1617674)
Supplement: Supplementary file 1 [file DataSheet1.docx]

Unveiling altered CD8 T-cell metabolism and homeostatic proliferation behind a low CD4/CD8 ratio in ART-suppressed HIV individuals with normal CD4 recovery

*Garrido-Rodríguez V. et al.*

# Supplementary Material

## Supplementary Tables

| Supplementary Table 1. Human primer sequences for qPCR. | | |
| --- | --- | --- |
| Target | | **Primer** |
| ASCT2 | Forward | CTACTTCCTCTTCACCCGCA |
|  | Reverse | GATGAAACGGCTGATGTGCT |
| GLUT1 | Forward | GGCCATCTTTTCTGTTGGGG |
|  | Reverse | CCAGCAGGTTCATCATCAGC |
| GDH | Forward | CTCCAGACATGAGCACAGGTGA |
|  | Reverse | CCAGTAGCAGAGATGCGTCCAT |
| HK1 | Forward | CTGCTGGTGAAAATCCGTAGTGG |
|  | Reverse | GTCCAAGAAGTCAGAGATGCAGG |
| LDH | Forward | ACCCAGTTTCCACCATGATT |
|  | Reverse | CCCAAAATGCAAGGAACACT |
| MCT1 | Forward | TACCTCCAGACTCTCCTGGC |
|  | Reverse | GTCCCCTCCGCAAAGTCTAC |
| SDH | Forward | GCATTATAACATGGGCGGCA |
|  | Reverse | GCCTGCATGACTCTTCGATG |
| PDH | Forward | GGATGGTGAACAGCAATCTTGCC |
|  | Reverse | TCGCTGGAGTAGATGTGGTAGC |
| Telomeres | Forward | GGTTTTTGAGGGTGAGGGTGAGGGTGAGGGTGAGGGT |
|  | Reverse | TCCCGACTATCCCTATCCCTATCCCTATCCCTATCCCTA |
| β-GLOBIN | Forward | ACACAACTGTGTTCACTAGG |
|  | Reverse | CAACTTCATCCACGTTCACC |

|  |  | | | |  |  |  |
| --- | --- | --- | --- | --- | --- | --- | --- |
| Supplementary Table 2. Metabolism-related genes mRNA relative expression. | | | | | | | |
| Cell subset | **Gene (relative expression)** | **R<0.8**  **(n=18)** | **R>1.2**  **(n=19)** | ***p*-value** | **N≤350**  **(n=18)** | **N>350**  **(n=19)** | ***p*-value** |
| CD4 T-cells | **MCT1** | 0.8[0.3-1.6] | 1.1[0.2-3.7] | 0.852 | 0.8[0.2-1.5] | 1.1[0.5-3.8] | 0.478 |
|  | **HK1** | 67[28.6-125.6] | 98.6[30.6-275.8] | 0.202 | 48.3[28.7-104] | 156[58.2-307.2] | *0.066* |
|  | **SDH** | 2.7[1.1-7.1] | 3.7[1-10.2] | 0.581 | 1.7[0.7-3.1] | 6[2.1-11.9] | **0.015** |
|  | **GDH** | 68.6[28.9-116.4] | 91.8[31.6-205.8] | 0.697 | 34.8[18.5-77.1] | 155[42-301.6] | **0.017** |
|  | **Glut1** | 31.5[8.1-66.2] | 22[8.3-60.6] | 0.283 | 18.5[7.3-33.8] | 51.7[8.4-115.7] | 0.105 |
|  | **PDH** | 16.5[7.7-45.5] | 20.4[7.7-97] | 0.363 | 11.1[4.2-20.1] | 54[18.2-111.9] | **0.010** |
|  | **ASCT2** | 7.8[3.4-29.2] | 9.1[2.3-35] | 0.709 | 5[2.8-14.3] | 15.9[3.5-48.4] | 0.260 |
|  | **LDH** | 153.2[60.3-620.4] | 132.5[77.3-1066.8] | 0.817 | 115.4[74.4-254] | 562.3[64.8-1424.6] | 0.217 |
| CD8 T-cells | **MCT1** | 1[0.2-5.4] | 0.9[0.3-1.8] | 0.451 | 1[0.8-1.7] | 0.6[0.1-4.4] | **0.041** |
|  | **HK1** | 66.7[30.1-245.8] | 37.5[11.6-114.6] | 0.547 | 59.1[29.9-107] | 49.9[7.8-117] | 0.371 |
|  | **SDH** | 2.1[1.1-13.9] | 3.8[1.2-8.6] | 0.370 | 2.9[1.4-9.4] | 3.5[1-13.1] | 0.423 |
|  | **GDH** | 57.5[19.9-183.9] | 37[17.1-62.7] | 0.126 | 57.5[33.2-125.2] | 50.2[7.2-167.7] | **0.009** |
|  | **Glut1** | 48[12.7-226.7] | 33.1[15.8-63.6] | 0.413 | 48[23.8-80.5] | 34.8[5.1-178.5] | **0.017** |
|  | **PDH** | 23.8[4.4-112.1] | 15.5[8.1-49.2] | 0.427 | 19.8[12.4-98.5] | 14.7[4.1-121.9] | **0.048** |
|  | **ASCT2** | 5.2[2.2-37.9] | 5.7[1.2-42.2] | 0.817 | 8[2.3-19.7] | 5.1[0.9-46.5] | 0.223 |
|  | **LDH** | 276.2[66-528.3] | 141[40.5-522.8] | 0.174 | 254.8[73.8-66.5] | 141[23.9-621.7] | **0.046** |
| Variables expressed as median [IQR] and represent mRNA relative expression *vs.* β-Globin; statistical comparisons between groups were performed using a non-parametric Mann-Whitney test. Abbreviations: MCT1, Monocarboxylate transporter; HK1, Hexoquinase 1; SDH, Succinate dehydrogenase; GDH, Glutamate dehydrogenase; Glut1, Glucose transporter 1; PDH, Pyruvate, dehydrogenase; ASCT2, Alanine Serine Cysteine transporter 2; LDH, Lactate dehydrogenase. Statistical significance is highlighted in bold. | | | | | | | |

## Supplementary Figures


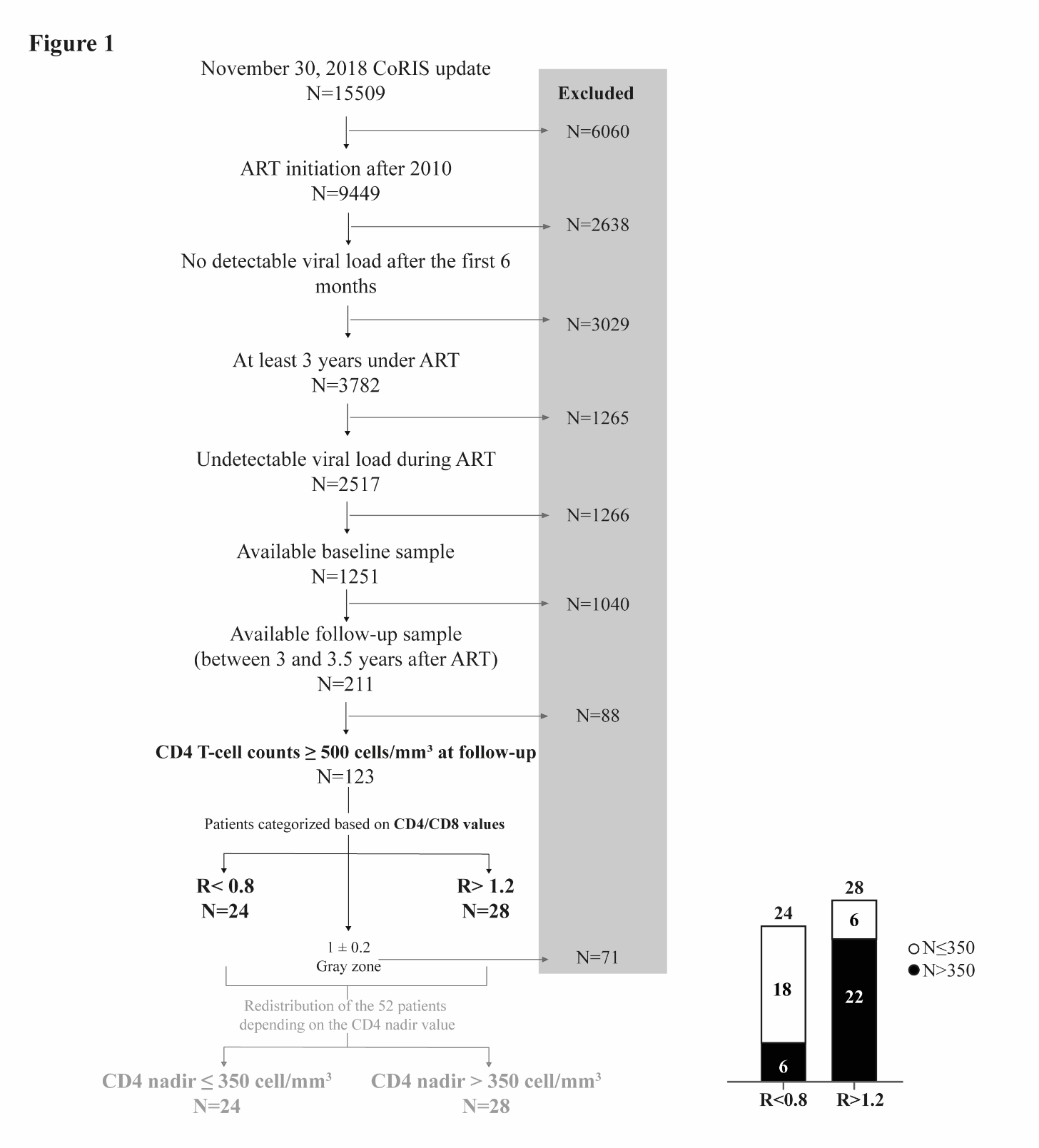


**Supplementary Figure 1. Flow chart and inclusion criteria.** We selected patients from the November 30, 2018 updated CoRIS database. We only selected those whose CD4/CD8 T-cell ratio was <0.8 or >1.2, , corresponding to 1±0.2 CD4/CD8 ratio values from the eligible patients, to avoid mixed phenotypes. Final selected patients were then reclassified for a secondary analysis according to their nadir-CD4 in ≤350 or >350 cell/mm^3^, since 350 cells/mm^3^ is a critical threshold for AIDS diagnosis, for monitoring treatment interruptions and also for identification of late-presentation. The number of subjects overlapping in comparison groups is represented in a cumulative bar graph as indicated in the legend.

# Annex

## Participant centers and collaborators of CoRIS and HIV Biobank

**CoRIS Executive committee:**

Santiago Moreno, Inma Jarrín, David Dalmau, M Luisa Navarro, M Isabel González, Federico Garcia, Eva Poveda, Jose Antonio Iribarren, Félix Gutiérrez, Rafael Rubio, Francesc Vidal, Juan Berenguer, Juan González, M Ángeles Muñoz-Fernández.

**CoRIS Coordination Unit**

Inmaculada Jarrín, Cristina Moreno, Marta Rava, Rebeca Izquierdo, Cristina Marco, Teresa Gómez-García.

**BioBanK HIV Hospital General Universitario Gregorio Marañón**

Mª Ángeles Muñoz-Fernández, Roxana Juárez.

**Hospital General Universitario de Alicante (Alicante)**

Joaquín Portilla, Irene Portilla, Esperanza Merino, Gema García, Iván Agea, José Sánchez-Payá, Juan Carlos Rodríguez, Livia Giner, Sergio Reus, Vicente Boix, Diego Torrus, Verónica Pérez, Julia Portilla, Héctor Pinargote.

**Hospital Universitario de Canarias (San Cristóbal de la Laguna)**

María Remedios Alemán, Ana López Lirola, Dácil García, Felicitas Díaz-Flores, M Mar Alonso, Ricardo Pelazas, María Inmaculada Hernández, Lucia Romero, Abraham Bethencourt, Daniel Rodríguez.

**Hospital Universitario Central de Asturias (Oviedo)**

Víctor Asensi, María Eugenia Rivas-Carmenado, Rebeca Cabo Magadan, Javier Díaz-Arias

**Hospital Universitario 12 de Octubre (Madrid)**

Federico Pulido, Rafael Rubio, Otilia Bisbal, M Asunción Hernando, David Rial, María de Lagarde, Adriana Pinto, Laura Bermejo, Mireia Santacreu, Roser Navarro, Juan Martín Torres.

**Servicio de Enfermedades Infecciosas. Hospital Universitario Donostia. Instituto de Investigación BioDonostia (Donostia-San Sebastián)**

José Antonio Iribarren, M José Aramburu, Xabier Camino, Miguel Ángel Goenaga, M Jesús Bustinduy, Harkaitz Azkune, Maialen Ibarguren, Xabier Kortajarena, Ignacio Álvarez-Rodriguez, Leire Gil, Francisco Carmona-Torre, Ana Bayona Carlos, Maialen Lekuona Sanz.

**Hospital General Universitario De Elche (Elche)**

Félix Gutiérrez, Catalina Robledano, Mar Masiá, Sergio Padilla, Araceli Adsuar, Rafael Pascual, Marta Fernández, Antonio Galiana, José Alberto García, Xavier Barber, Javier García Abellán, Guillermo Telenti, Lucía Guillén, Ángela Botella, Paula Mascarell, Mar Carvajal, Alba de la Rica, Carolina Ding, Lidia García-Sánchez, Nuria Ena, Leandro López, Jennifer Vallejo, Nieves Gonzalo-Jiménez, Montserrat Ruiz, Christian Ledesma, Santiago López, María Espinosa, Ana Quiles, María Andreo.

**Hospital General Universitario Gregorio Marañón (Madrid)**

Juan Carlos López Bernaldo de Quirós, Isabel Gutiérrez, Juan Berenguer, Margarita Ramírez, Paloma Gijón, Teresa Aldamiz-Echevarría, Francisco Tejerina, Cristina Diez, Leire Pérez, Chiara Fanciulli, Saray Corral.

**Hospital Universitari de Tarragona Joan XXIII (Tarragona)**

Joaquín Peraire, Anna Rull, Anna Martí, Consuelo Viladés, Beatriz Villar, Lluïsa Guillem, Montserrat Olona, Graciano García-Pardo, Frederic Gómez-Bertomeu, Verónica Alba, Silvia Chafino, Alba Sánchez.

**Hospital Universitario y Politécnico de La Fe (Valencia)**

Marta Montero, María Tasias, Eva Calabuig, Miguel Salavert, Juan Fernández, Rosa Blanes.

**Hospital Universitario La Paz/IdiPAZ (Madrid)**

Juan González-García, Ana Delgado-Hierro, José Ramón Arribas, Víctor Arribas, José Ignacio Bernardino, Carmen Busca, Joanna Cano-Smith, Julen Cardiñanos, Juan Miguel Castro, Luis Escosa, Iker Falces, Pedro Herranz, Víctor Hontañón, Alicia González-Baeza, M Luz Martín-Carbonero, Mario Mayoral, Rafael Micán, Rosa de Miguel, Rocío Montejano, Mª Luisa Montes, Luis Ramos-Ruperto, Berta Rodés, Talía Sainz, Elena Sendagorta, Eulalia Valencia, M del Mar Arcos, Alejandro de Gea Grela, Carlos Oñoro López.

**Hospital Universitari Mutua Terrassa (Terrassa)**

David Dalmau, Marina Martinez, Angels Jaén, Mireia Cairó, Javier Martinez-Lacasa, Roser Font, Laura Gisbert.

**Hospital Universitario de La Princesa (Madrid)**

Ignacio de los Santos, Alejandro de los Santos, Lucio García-Fraile, Enrique Martín, Ildefonso Sánchez-Cerrillo, Marta Calvet, Ana Barrios, Azucena Bautista, Carmen Sáez, Marianela Ciudad, Ángela Gutiérrez, **María Aguilera García.**

**Hospital Universitario Ramón y Cajal (Madrid)**

Santiago Moreno, Santos del Campo, José Luis Casado, Fernando Dronda, Ana Moreno, M Jesús Pérez, Sergio Serrano-Villar, Mª Jesús Vivancos, Javier Martínez-Sanz, Alejandro Vallejo, Matilde Sánchez-Conde, José Antonio Pérez-Molina, José Manuel Hermida, Erick de La Torre Tarazona, Elena Moreno, Laura Martín Pedraza, Claudio Díaz García, Jorge Díaz, Alejandro García, Raquel Ron.

**Hospital General Universitario Reina Sofía (Murcia)**

Enrique Bernal, Antonia Alcaraz, Joaquín Bravo, Ángeles Muñoz, Cristina Tomás, Eva Oliver, David Selva, Eva García, Román González, Elena Guijarro, Rodrigo Martínez, María Dolores Hernández.

**Hospital Universitario Clínico San Cecilio (Granada)**

Federico García, Clara Martínez, Leopoldo Muñoz Medina, Marta Álvarez, Natalia Chueca, David Vinuesa, Adolfo de Salazar, Ana Fuentes, Emilio Guirao, Laura Viñuela, Andrés Ruiz-Sancho, Francisco Anguita, Naya Faro, José Peregrina, Lucia Chaves, Marta Illescas, Valme Sánchez.

**Centro Sanitario Sandoval (Madrid)**

Jorge Del Romero, Montserrat Raposo, Carmen Rodríguez, Teresa Puerta, Juan Carlos Carrió, Mar Vera, Juan Ballesteros, Oskar Ayerdi, Begoña Baza, Eva Orviz.

**Hospital Universitario Son Espases (Palma de Mallorca)**

Melchor Riera, María Peñaranda, M Angels Ribas, Antoni A. Campins, Mercedes Garcia-Gazalla, Francisco J Fanjul, Javier Murillas, Francisco Homar, Helem H Vilchez, Luisa Martin, Antoni Payeras.

**Hospital Universitario Virgen de la Victoria (Málaga)**

Jesús Santos, María López, Cristina Gómez, Isabel Viciana, Rosario Palacios.

**Hospital Universitario Virgen del Rocío (Sevilla)**

Luis Fernando López-Cortés, Nuria Espinosa, Cristina Roca, Silvia Llaves.

**Hospital Universitario de Bellvitge (Hospitalet de Llobregat)**

Juan Manuel Tiraboschi, Arkaitz Imaz, María Saumoy.

**Hospital Costa del Sol (Marbella)**

Julián Olalla, Javier Pérez, Alfonso del Arco, Javier de la Torre, José Luis Prada.

**Hospital General Universitario Santa Lucía (Cartagena)**

Onofre Juan Martínez, Lorena Martinez, Francisco Jesús Vera, Josefina García, Begoña Alcaraz, Antonio Jesús Sánchez Guirao.

**Complejo Hospitalario Universitario a Coruña (CHUAC) (A Coruña)**

Álvaro Mena, Berta Pernas, Pilar Vázquez, Soledad López, Brais Castelo.

**Hospital Universitario Virgen de la Arrixaca (El Palmar)**

Carlos Galera, Marian Fernández, Helena Albendin, Antonia Castillo, Asunción Iborra, Antonio Moreno, M Angustias Merlos, Inmaculada Chiclano.

**Hospital Universitario Infanta Sofía (San Sebastián de los Reyes)**

Inés Suarez-García, Eduardo Malmierca, Patricia González-Ruano, M Pilar Ruiz, José Francisco Pascual, Luz Balsalobre, Ángela Somodevilla.

**Hospital Universitario Príncipe de Asturias (Alcalá de Henares)**

José Sanz, Alberto Arranz, Cristina Hernández, María Novella.

**Hospital Clínico Universitario de Valencia (Valencia)**

María José Galindo, Sandra Pérez Gómez, Ana Ferrer.

**Hospital Reina Sofía (Córdoba)**

Antonio Rivero Román, Inma Ruíz, Antonio Rivero Juárez, Pedro López, Isabel Machuca, Mario Frias, Ángela Camacho, Ignacio Pérez, Diana Corona, Javier Manuel Caballero.

**Hospital Universitario Severo Ochoa (Leganés)**

Rafael Rodríguez-Rosado Martinez-Echevarría, Rafael Torres.

**Hospital Universitario Virgen de Valme (Sevilla)**

Juan Macías Sánchez, Pilar Rincón, Luis Miguel Real, Anais Corma, Alejandro González-Serna.

**Hospital Álvaro Cunqueiro (Vigo)**

Eva Poveda, Alexandre Pérez, Luis Morano, Celia Miralles, Antonio Ocampo, Guillermo Pousada, María Gallego, Jacobo Alonso, Inés Martínez.
